# Perspectives and Experiences of Policy Makers, Researchers, Health Information Technology Professionals, and the Public on Evidence-Based Health Policies: Protocol for a Qualitative Study

**DOI:** 10.2196/16268

**Published:** 2020-12-17

**Authors:** Anastasia Mallidou, Dzifa Dordunoo, Elizabeth Borycki, Andre Kushniruk, Kirsten Sadeghi-Yekta, Julie Fraser, Sirisha Asuri

**Affiliations:** 1 School of Nursing University of Victoria Victoria, BC Canada; 2 School of Health Information Science University of Victoria Victoria, BC Canada; 3 Theatre Department University of Victoria Victoria, BC Canada; 4 Professional Regulatory Practice Department Fraser Health Vancouver, BC Canada; 5 Primary Care Division BC Ministry of Health Victoria, BC Canada

**Keywords:** evidence-based health policy, knowledge translation, transparency, policy makers, researchers, knowledge producers, health information technology

## Abstract

**Background:**

Evidence-based health policy (EBHP) development is critical to the judicious use of public funds. EBHPs increase transparency, accountability, effectiveness, and efficiency of policies. Encouraging collaboration between researchers or knowledge producers and policy makers is important because both communities have distinct professional cultures, resulting in them working separately without understanding each other. Knowledge sharing is a complex process that requires understanding of cultural aspects that may reduce cultural differences and increase the use of common language. Health information technology (HIT) is a useful tool to increase knowledge translation, which may result in the transparent use of evidence and networking in developing EBHPs. Our vision is to leverage HIT tools for a better health system that includes digitalized, open source, evidence-based, and transparent ways for collaboration and development of robust mechanisms and for sharing of synthesized evidence with knowledge user–friendly forms.

**Objective:**

The aim of this study is to develop a conceptual framework on Knowledge translation and health Information Technology for Transparency (KhITT) in policy making and EBHPs (ie, the KhITT framework). The framework will be informed by the views of four key stakeholder groups (ie, policy makers, knowledge producers, HIT professionals, and the public) toward EBHP. The informants may also describe practices that demonstrate the EBHP development process and suggest technology platforms to enable this process.

**Methods:**

We propose an exploratory, descriptive qualitative study to take place in British Columbia, Canada, using in-depth semistructured interviews. To ensure data saturation and trustworthiness, we will use a nonprobability, purposive snowball sample of up to 15 eligible participants in each of the four stakeholder groups. We will analyze the data using content analysis.

**Results:**

The KhITT framework focuses on various stakeholders’ perspectives to better understand their perceived needs and priorities in identifying issues with EBHP, in order to make informed recommendations. Ethics approval has been obtained by the harmonized Behavioural Research Ethics Board at the University of British Columbia. We anticipate that we will complete data collection and analysis by December 2020. Preliminary results will be published in summer 2021.

**Conclusions:**

Our ultimate goal of this study is to develop a conceptual framework and describe the technology platforms that would enable the EBHP process. We anticipate that our rigorous content analysis will be able to produce insights and themes that are able to address our objectives, contribute to an in-depth understanding of the EBHP process within British Columbia, highlight all influential factors, explicitly disseminate and communicate the study results, identify issues with EBHP and provide informed recommendations to address them, and enhance efforts toward transparent EBHPs.

**International Registered Report Identifier (IRRID):**

PRR1-10.2196/16268

## Introduction

### Background

The movement for evidence-based health policy (EBHP) has made progress at all levels of government: local, provincial or state, and federal. Developing EBHPs is a critical way to ensure effective and efficient use of public funds and other scarce resources. In addition, using evidence-based methods in health policy development can increase transparency and accountability of these policies. Focus and efforts must align with innovative and effective products and services that will lead to stronger collaborations. Encouraging collaboration and innovative ways of policy making is important, especially when evidence is thin, research is limited, or when some public agencies lack the capacity, skills, knowledge, funding, commitment, and/or support of political leaders to integrate evidence into policy. A movement toward policy-based evidence, noted as found in Young [1], was depicted in *The New Yorker* magazine as a cartoon: a policy maker handed a paper to an advisor saying, “Here is my policy; go find some evidence based on it.” Health policy makers have their own priorities and processes that influence and have implications on health outcomes.

### Culture of Collaboration

In the literature, there is an ongoing debate between researchers and policy makers about EBHP, mainly because of a misunderstanding or a lack of a common definition of *evidence* [2,3] as well as a lack of agreement on what constitutes evidence across disciplines [4]. The communities of researchers have perspectives in the development of health policies that are distinct from those of policy makers, which result in these professional cultures working in separate “silos.” For example, Glazer and Karpati [5] described several cross-cultural differences in decision-making styles; however, it is unclear whether those different decision-making approaches are focused on scientific evidence. Evidence matters to policy making, which is political due to the trade-offs involved between multiple competing interests [6], but choosing the right evidence may politicize science (eg, misuse and cherry picking). On the other hand, knowledge producers suggest that synthesized scientific knowledge is the foundation of health policy. Other cultural differences and discrepancies between knowledge producers and policy makers include the definition of, and perceptions about, evidence and its validity and reliability, adequacy and interpretation of research findings, and the understanding of using evidence as part of the decision process. While discussions about these topics are frequent and inherent to policy debates that develop distrust and conflict between knowledge producers and policy makers [7], “collaboration between these groups, regardless of its complex and time-intensive process, requires trust and partnership” [8]. Both knowledge producers and policy makers need to resolve these differences, connect, communicate, understand each other, and collaborate in order to improve the process of policy making and to develop robust policy, since study designs, assessments of quality, and the ability of research to inform policy making varies by discipline. There is also a need to develop technology platforms that allow for the exchange of knowledge and information. Communication among knowledge producers and policy makers could promote shared and mutual understanding of evidence in each discipline and ways that evidence can be applied to other disciplines (eg, application of health informatics research findings to evidence-based policy-making activities) [4,9]. For example, more effective, efficient, and humane responses to disasters can be provided when key stakeholders’ perspectives are taken into account, community is engaged in the required discussions and collaborations, and strong evidence is available [10].

In Australia, evidence-generation partnerships and levels of collaboration between researchers and policy makers vary widely from minimal to coproduction, which all partners considered as a worthy goal with many benefits [11]. Furthermore, the main themes underpinning the challenges faced in health communication and participation among researchers and other stakeholders were culture and organizational structures, health professional attitudes and assumptions, lack of shared or overlapping knowledge of a domain area, and lack of shared understanding in the health sector. Therefore, setting priorities for knowledge-synthesis research, including evidence-based policy making, embraces interventions to enhance health professional education, to change health service and health professional cultures and attitudes, and to improve health service policies and standards [12].

### Data Visualization

Researchers also need to make their studies transparent and available to the public, including to policy makers; help people make sense of the data; and develop and share their study results using visual means (eg, data visualizations) for better understanding and uptake of key messages [13]. Data visualization, when aligned with the principles of trustworthiness and accessibility, supports decision making and *facilitates understanding* through three main ways. First, it effectively communicates data. Second, it provides people with the opportunity to explore, examine, analyze, and identify patterns within the data and to better understand large data sets. Finally, it encourages and affects user engagement via “consumption and production processes” [13] of visual data. Production of data visualization and its consumption have the power to change people’s minds and maybe change the world. Understanding data production, visualization, and consumption processes within certain contexts may unveil the entanglement of, and the power within, the data and may contribute to the political impact for doing good, guidelines for good practice, and the limits of data visualization within complex situations [14].

### Evidence-Based Health Policy

Despite important advances, rigorous evaluation requirements, and other evidence-based approaches in policy making, the majority of policies are rarely based on rigorous evidence [15]. Evidence-based policy frameworks are usually not intended to be prescriptive but are intended to emphasize the multidimensionality of the policy-making process, the causal relationships between the different dimensions, the indicators to measure selected dimensions, the determinants of poor outcomes, the assessment of the policy environment, the interplay among policies and social norms, the evaluation of the impact and cost-effectiveness of interventions and programs, the approaches to support people’s voices, and the inclusion of relevant evidence in policy-making processes [16]. The political and institutional context is one of the most important issues around evidence-based policy [17,18]. Within this context, the process of policy making and the nature of information and evidence used varies according to key personnel approaches, since individuals negotiate the concept of socially constructed evidence (eg, common sense, expert opinion, and filters to transferred evidence) [18]. Therefore, study and interpretation of locally tailoring contextual complexities of policy-making practices and processes may be more useful than a one-size-fits-all evidence-based policy framework. These frameworks may provide a better understanding of processes to influence and develop meaningful collaborations between knowledge producers and policy makers [19]. In addition, capacity building, one of the identified barriers for collaboration between knowledge producers and policy makers, may be overcome by involving policy makers in conducting research and by involving knowledge producers in developing policies within a certain context [18].

### The Preliminary Conceptual Framework

In this environment, we developed a vision for an ideal health system where everything will be digitalized, open source, evidence based, and transparent. That health system may lead to fewer adverse events; better patient, provider, and system outcomes; greater innovation; and less ineffective use of resources (eg, monetary and human). To achieve this ideal health system, we need to focus on three main principles:

1. Collaboration between knowledge producers and policy makers for sharing the understanding of an issue and accepting each other’s knowledge, evidence base, and cultural manifestations.
2. Development of robust mechanisms at an organizational level for synthesizing existing knowledge on a policy maker’s interesting topic.
3. Communication and sharing of synthesized evidence in forms that are friendly to policy makers [20].

To actualize this vision, we propose a conceptual framework called the Knowledge translation and health Information Technology for Transparency (KhITT) in policy making and EBHPs (ie, the KhITT framework). A preliminary conceptual framework was recently developed and published [21]. Our idea for a conceptual framework to connect researchers and policy makers originated during the development of a course for graduate students at the University of Victoria School of Nursing: *Evidence-Based Health Policy*. We argue that health care professionals need to be aware and contribute to the health policy–making process. We anticipate further development of the KhITT conceptual framework to provide insights and ways for improving collaboration between researchers and policy makers for the development of health policies that are transparent and evidence based. The KhITT framework builds upon the strong commitment of knowledge producers and policy makers to finding effective solutions and encourages the use of technology that supports the development of innovative health policies. For example, Tran and colleagues’ [22,23] work is an example of the successful use of technology to predict consequences of future scenarios and to inform policy making that has resulted in the development of innovative health policies to bring computerized order entry and electronic medical records into health settings. This area of research on the impact of information technology interventions in health and the decision-making process is underexplored, especially for outcomes commonly required by policy makers and government [24]. Collaboration between policy makers and knowledge producers would enhance efforts to set and realize high-reaching goals.

The KhITT framework aims to *support* strong relationships between knowledge producers and policy makers and to *inspire* individuals and the public to commit to excellence in engaging communities and stakeholders toward transparent, system-based knowledge dissemination and EBHPs. The KhITT framework is comprised of recommendations that are organized within three domains: structure, reference and guide, and capacity building. Specifically, the goals of these domains are as follows:

1. To provide a structure for understanding each other’s perspective, improving communication, and strengthening coordination efforts for effective solutions.
2. To serve as a reference and guide for strategic choices and for setting priorities and strategies in incorporating evidence into health policy innovation.
3. To nurture existing and emerging knowledge producers and policy makers who are interested in promoting EBHPs by exploring ways to use health information technology (HIT) (ie, capacity building).

This framework emphasizes the important contribution of HIT as a useful tool to increase knowledge translation and transfer via communication among knowledge producers and policy makers that may result in the transparent use of evidence and networks in developing EBHPs [25]. Sharing knowledge is a complex process that also incorporates sharing of cultural aspects, which in turn may reduce the cultural differences between knowledge producers and policy makers and may increase the use of common language [25]. The development of information and communications technologies has dramatically changed the context and the way we accumulate evidence and make policies. Access to evidence, including academic and grey literature, used to depend on consultants or other persons who had access to libraries and who knew how to navigate scholarly journals. The development of searchable online databases changed the way policy makers access evidence. However, we need more efforts to enhance collaboration between researchers; knowledge users, including policy makers; and HIT professionals and researchers to develop EBHPs. There is a need to develop technology platforms that allow for the exchange of knowledge and the development of shared and mutual understanding across disciplines regarding what is good-quality evidence and how that evidence could be interpreted and effectively applied to policy-making tasks and activities in another discipline [26,27]. HIT is most commonly used in hospitals and nonhospital-based clinics to support clinical decision making [24]. We need to evaluate the impact of HIT interventions in the health sector beyond this area.

### Purpose

The purpose of this study is to develop a conceptual framework on KhITT in policy making and EBHPs (ie, the KhITT framework). The specific objectives to achieve this aim are as follows:

1. Explore and better understand key stakeholders’ (ie, policy makers, knowledge producers, HIT professionals, and the public) different approaches on EBHPs.
2. Capture those different approaches within three main categories—perceptions, perspectives, and experiences—to better understand participant expectations and insights from each stakeholder group and to incorporate them into the KhITT conceptual framework.
3. Describe practices used for demonstrating the EBHP development.
4. Describe the technology platforms that would enable the EBHP development process.

### Definitions

For the purposes of this study, we define the relevant specific terms as follows:

1. Knowledge translation, as defined by the Canadian Institutes of Health Research, is “a dynamic and iterative process that includes synthesis, dissemination, exchange and ethically sound application of knowledge to improve the health of Canadians, provide effective health services and products and strengthen the health care system” [28]. This process takes place within a complex system of interactions between knowledge producers and knowledge users, including policy makers, which may vary in intensity, complexity, and level of engagement depending on the nature of knowledge [29].
2. HIT is defined as the technology applied to the health sector that supports information management across computerized systems and improves all aspects of health care (eg, safety, effectiveness, timeliness, equity, and efficiency). Open access sources of information embody radical change, make HIT broadly available, and provide a forum for sharing information and knowledge toward software development and democratic action. This open communication model could start political discourse among knowledge producers and policy makers.
3. Transparency is defined as openness, accountability, obligation, and honesty to share information and knowledge with the public.
4. Policy making is defined as a messy iterative process with various opportunities to incorporate evidence and strengthen decisions [30].
5. EBHP is defined as the integration of individual (ie, policy makers) professional expertise, experience, and practice with the best available research findings in the context of specific preferences and values. We adopted this description of EBHP by paraphrasing Sackett and associates’ [31] definition of evidence-based practice, including policy making.
6. Perception is defined as a way of understanding, interpreting, or thinking about something through the fundamental senses. Perception depends on complex effortless functions of the nervous system and is influenced by experiences, feelings, and thoughts [32,33].
7. Perspective is defined as a point of view, a particular way of viewing things that depends on one’s attitude, experience, and personality.

## Methods

### Design, Settings, and Sample

To achieve the aim and objectives of this study, we propose an exploratory, descriptive, qualitative study design to take place in British Columbia, Canada. We will collect data from policy-making centers (eg, the Ministry of Health), universities (eg, the University of Victoria and University of British Columbia), public areas (eg, malls, cafeterias, and personal contacts), and HIT workplaces (eg, health authorities and the Ministry of Health) using a nonprobability, purposive snowball sample of four stakeholder groups: knowledge producers, policy makers, HIT professionals, and the public. Specifically, in Victoria and Vancouver, we will carefully target, invite for participation, recruit, and interview up to 15 participants from each of the following stakeholder groups:

1. Researchers in academic institutions, whose work is relevant to health policies, including HIT researchers, to ensure that evidence from HIT research is introduced, due to the differences in how evidence is generated through research and how it is used in practice.
2. Policy makers at all levels of governance (ie, local, provincial, and federal) whose work focuses on health policies.
3. HIT professionals, such as electronic health record managers, developers, or analysts. We hypothesize that there will be a disconnection between HIT researchers and HIT practitioners in terms of perspectives and the definition and use of evidence to drive policy and decision making.
4. Citizens (ie, the public), regardless of gender, age, ethnicity, class, education, socioeconomic status, position, or other demographic characteristics. We will invite individuals older than 18 years of age who are interested in EBHP to participate in the research project.

We will start recruiting study participants at the University of Victoria (ie, researchers), the Vancouver Island Health Authority (ie, HIT professionals), and the provincial government located in Victoria (ie, policy makers and the public). We are focusing on those stakeholder groups because we expect each group of participants to provide different perceptions, perspectives, and experiences relevant to EBHP and the EBHP development process, which we want to capture and incorporate into the KhITT conceptual framework for a better understanding of their expectations and insights.

We estimate that the number of participants we plan to recruit will provide the needed data saturation [34,35]. Data saturation is reached when (1) there is adequate information to replicate the study [36,37], (2) the ability to obtain additional new information or new themes has been attained [38], and (3) further coding is no longer feasible [38].

To ensure that data saturation has been reached in our study (ie, no new themes), we will construct a saturation grid, where major topics will be listed on the vertical axis and conducted interviews on the horizontal axis; at least two research team members will conduct coding of transcripts independently [39]. In addition, we may apply the mathematical model developed by Tran and colleagues [40] to compute the theme accumulation curve and the local slope of the curve at the point of data analysis and our chosen stopping criterion.

### Instrument

We will collect the data using in-depth face-to-face or telephone-based semistructured interviews [41]; the interview format will depend on participant availability and preference, and interviews will last about 60 minutes for each participant. All interviews will be recorded on a digital tape (ie, face-to-face interviews) or electronically (ie, interviews via telephone) with participants’ informed consent.

For the interviews, we will develop and use our own semistructured instrument and guide (see example questions in Multimedia Appendix 1). The interview guide will cover the general and main study topics, provide the setting to encourage participants to share their perceptions and experiences, and focus on the following key areas:

1. Participant background information.
2. Definitions (eg, policy process and EBHPs).
3. Main themes that explore participants’ knowledge, perceptions, perspectives, and experiences on methods and/or practices used to identify EBHPs.
4. Follow-up questions, including prompts and stimuli aimed at following respondents’ answers and in-depth investigation of any issues raised.

Prior to data collection, we will test and pilot the interview instrument and guide with several eligible participants, and we will revise and adapt it accordingly to ensure that the interview questions are meaningful to the respondents’ backgrounds [42]. In addition, we will adjust the instrument based on formative or ongoing data analysis [43].

### Data Collection Process

We will recruit study participants through professional lists, email address listservs, personal contacts, and advertisements in social media accounts (ie, study Twitter and Facebook accounts), local newspapers (eg, the *Times Colonist*), and public places (eg, restaurants and malls). Upon ethics approval, a recruitment posting will be sent to the social media accounts of all known British Columbia policy makers, knowledge producers, and HIT professionals, including links to the study materials. We will ask eligible participants (ie, policy makers, knowledge producers, HIT professionals, and the public) to pass our study information (ie, invitation to participate and information letter) to any other interested participants. Research assistants will approach potential eligible participants in all stakeholder groups, inform them about the study, and answer questions for clarification in person, via email, or via phone. Research assistants will also provide hard copies of the study materials to interested individuals and request their signed informed consent to participate in the study. Then, research assistants will arrange a face-to-face or telephone-based interview at a time and place that is convenient to each participant. At the beginning of the interview, research assistants will again request verbal consent and explain the interview process. During the audio-recorded interviews, pseudonyms or numerical identifiers will be used to protect the anonymity and confidentiality of the participants. In addition, research assistants will make *field notes* during and immediately after each interview about observations, thoughts, and ideas about the interview to help the data analysis process.

### Data Analysis

We will analyze the collected data following the steps of a content analysis and describe the emerged themes and patterns about the EBHP process. First, we will deidentify (ie, anonymize) the data for study participant anonymity and confidentiality. Then, we will transcribe verbatim the collected data (ie, recorded interviews). The lead researcher will transcribe two to three interviews to inform the analytic process; a professional transcription agency, or the research assistants themselves, will transcribe the remaining audio files. Finally, we will manage, process, and store the deidentified data files in a secure, digital, password-protected form in the principal investigator’s locked office using the University of Victoria’s technology and servers (eg, lockable computer systems with encryption protection).

According to Braun and Clarke’s [41] approach, to generate a good thematic analysis we will include the following steps:

1. Transcription and review of transcripts for accuracy.
2. Open coding by at least two researchers independently; themes to be checked against each other and against the original data for internal coherence, consistency, and distinctiveness.
3. Development of an initial codebook in agreement with the research team.
4. Analysis of the remaining interviews based on the initial codebook may follow, such as creating new codes or refining of existing ones, developing themes and subthemes to identify similarities and differences in the interviews, developing categories of meanings in order to group themes into broader (ie, more abstract) concepts, and establishing a good balance between analytic narrative and illustrative extracts.
5. Thematic map to be developed based on the identified themes, subthemes, and categories as well as their relationships.

During our thematic data analysis, which is guided by Lincoln and Guba [44], we will ensure trustworthiness of our study by addressing the following tenets:

1. Credibility, which refers to the establishment of “confidence in the truth of the findings.”
2. Transferability, which refers to the extent to which the study findings are applicable in other contexts (ie, generalizability).
3. Dependability, which refers to whether the research question is clear and logically associated with the study purpose and design, as well as to the consistency of findings in a replicated study.
4. Confirmability, which refers to objectivity or neutrality; that is, the degree to which the study findings are determined by the participants and clearly derived from the data and not by the researcher’s “biases, motivations, interests, or perspectives.”
5. Reflexivity, which refers to the researcher’s own conceptual lens, explicit and implicit assumptions, preconceptions, and values that may affect research decisions during all phases of a study [45].

Throughout data collection and analysis, we will ensure traceability and verification as part of the study quality using the following strategies to establish trustworthiness [46-48]:

1. Researcher triangulation, for credibility, transferability, and dependability. That is, engagement of multiple researchers, as well as graduate and undergraduate students, to incorporate their unique insights into the interpretation of findings; planned debriefing sessions among the research team members to provide a sound space on elaborating different study ideas and interpretations; and reporting the background and context descriptions of our study findings from the study participants.
2. Participant validation, for credibility and confirmability, by seeking respondents’ reflections on the transcripts and interpretation of results.
3. Reflexive journal to document researchers’ thoughts and reflections throughout the research process and for discussing any emerging issues.
4. Other general strategies, such as storing raw data systematically, recording the rationale and justification of methodological and analytical choices, transparently describing all the study steps, documenting detailed notes about the development and hierarchies of themes, and establishing consensus on themes.

Additionally, we may provide the opportunity to all interested stakeholders to have free access to the research process by developing the KhITT study website and data visualizations to better understand, and make better use of, our study findings.

### Patient and Public Involvement

Our research team includes policy makers, researchers, HIT experts, and members of the public as key informants for better understanding their perceived needs and priorities in identifying issues with EBHP, in order to make informed recommendations. Together, we designed the proposed study and we will collaborate closely in the data collection and in the interpretation, dissemination, and diffusion of study findings. In addition, we will establish an advisory group of policy makers, HIT professionals and researchers, and citizens, who will support the research process, provide input and insights into the data analysis and interpretation of the findings, and contribute to the dissemination plan. The advisory group will meet on a regular basis during the study.

### Ethics

We have already obtained ethics approval by the harmonized Behavioural Research Ethics Board at the University of British Columbia using their online research administration tool. For the duration of the study, we incorporated procedures that intended to protect participant anonymity and confidentiality.

### Data Sharing Statement

Supplementary and raw data will be placed online, made publicly available, and linked to the University of Victoria repository.

### Dissemination Plan

At the completion of the study, we will present the findings at local, national, and international scientific conferences; professional events for stakeholder groups, including interviewees if they are interested in and willing to share their contact information, for better stakeholder engagement; and our professional websites, where we will provide data visualizations. Findings will be presented in a summarized form with no identifying information. We will also publish the results in peer-reviewed journals, professional and lay magazines, and the study website.

## Results

The KhITT framework focuses on various stakeholders’ perspectives to better understand their perceived needs and priorities in identifying issues with EBHP, in order to make informed recommendations. Ethics approval has been obtained by the harmonized Behavioural Research Ethics Board at the University of British Columbia. Currently, we are recruiting study participants and collecting their consent forms. We anticipate that we will complete data collection and analysis by December 2020. Preliminary results will be published in summer 2021.

## Discussion

### Principal Findings

The KhITT conceptual framework focuses on the routine enhancement of engagement, flexible accessibility, and strong relationships among knowledge producers and policy makers as key factors influencing transparent policy making [49]; the framework also focuses on addressing and teasing out disciplinary differences and their impact on policy makers’ decisions due to lack of experience in a particular domain area. We are also interested in determining research quality between clinical research, policy research, health services research, and health informatics research. For example, health informatics researchers value mixed methods studies more than randomized clinical controlled trials. Evidence from mixed methods studies better informs the practice of health informatics than evidence from clinical controlled trials. We believe that health services research and policy research suffer from the same issue: study quality varies based on prior disciplinary work. What works for one discipline is not necessarily transferable to another, in terms of research quality. This phenomenon may influence cross-disciplinary education. In addition, we will examine and take into consideration context as a factor that might influence evidence use in policy making [50], and we will illuminate barriers and facilitators in making EBHPs based on the various participant perspectives. Finally, the potential development of an electronic tool, emphasizing the important contribution of HIT, can support and increase the exchange of evidence among knowledge producers and policy makers.

### Impact of the KhITT Framework

The KhITT framework fills a gap in the literature, since there are not a lot of technology policy frameworks. There is a need to delineate policy to help guide implementations, as there have been issues with the success of health care information technology implementations worldwide. We hope that the developing conceptual framework, which includes an electronic platform, will be a useful tool for researchers and policy makers to practice closer collaboration and to influence each other’s world and work. In addition, we anticipate that the KhITT study findings will have an impact on changing behaviors and enhancing use of evidence in health policy. Specifically, we expect that the use of our research findings will serve as a tool that may help with the following [51,52]:

1. Inform and enlighten policy making and change knowledge, awareness, attitudes, or opinions about the process of making decisions and health policies, but not necessarily action (ie, indirect or conceptual use).
2. Make specific decisions and policies or interventions by directly applying research in a useable form, such as a brief or a protocol (ie, direct or instrumental use).
3. Legitimate a position, win an argument, make a case, or persuade those in decision-making positions, in order to change.

### Conclusions

The ultimate goal of the proposed KhITT framework is to develop a conceptual framework and describe the technology platforms that would enable the EBHP development process. We anticipate that our rigorous content analysis will be able to produce insights and themes that are able to address our objectives, contribute to an in-depth understanding of the EBHP process within British Columbia, highlight all influential factors, explicitly disseminate and communicate the study results, identify issues with EBHP and provide informed recommendations to address them, and enhance efforts toward transparent EBHPs.

## Appendix

Multimedia Appendix 1 Interview questions.

## Abbreviations

EBHP: evidence-based health policy
HIT: health information technology
KhITT: Knowledge translation and health Information Technology for Transparency
