# Supplementary material for: Perspectives and Experiences of Policy Makers, Researchers, Health Information Technology Professionals, and the Public on Evidence-Based Health Policies: Protocol for a Qualitative Study
Source: JMIR Res Protoc. 2020 Dec 17;9(12):e16268. doi: 10.2196/16268 (PMC7775201; doi:10.2196/16268)
Supplement: Multimedia Appendix 1 [file resprot_v9i12e16268_app1.docx]

Multimedia Appendix 1

Interview Guide and Sample Questions

Thank you for participating in this study and accepting to beeing interviewed today about your perceptions, perspectives, and experiences relevant to EBHPs and EBHP process. You have been invited to participate because of your interest and/or extensive experience in EBHP. During this interview, we are hoping to learn EBHP process and EBHPs.

Specifically,

What is the process of policy-making in health, in your experience and/or understanding?

What *are* the sources of information for a health policy, from your point of view?

What *should be* the sources of information for a health policy?

What is evidence, to your understanding/knowledge?

How would you describe the use of evidence in the policy-making process in health?

How would you describe an evidence-based health policy (EBHP), according to your understanding and/or experience?

1. What factors do you think increase the use of evidence in policy-making?

What are some of the barriers policy makers experience in seeking comphrehensive evidence?

Can you give me an example of a time you felt that a policy (or policy-making process) *was* based on evidence, to your understanding or experience?

How was/would be a situation different when a policy (or policy-making process) *was not* based on evidence as you would expected to be?

What are any examples/experiences of non-EBHP activities or process?

What are any practices that may demosnstrate EBHP process and/or policies?

Please describe in as much detail as you wish to share your experience of EBHP process and policy itself.

How do you understand a collaboration among researchers and policy-makers?

How can collaborations and partnerships between researchers and policy-makers be initiated? Identify specific facilitators.

What are your insights toward a collaboration among researchers and policy-makers?

What are your expectations from this collaboration?

What do researchers and policy-makers actually deliver?

How does/would a partnership among researchers and policy-makers work?

How would you describe a successful partnership among researchers and policy-makers?

1. What factors do you think have led to a successful partnership among researchers and policy-makers?
2. What are some common barriers to a successful partnership that you have come across?
3. What factors do commonly drive suboptimal partnerships?
4. What are some common indicators that a partnership has not worked?

How would you describe the role of health information technology (HIT) in the policy-making process?

What would be any technology platforms that would enable EBHP process?

How would you describe collaboration among researchers and policy-makers using technology?

Thank you for your time!
